# Integrating Radiology and Metabolic Risk: DEXA-Based Characterization of Bone Health in Type 2 Diabetes

**DOI:** 10.3390/metabo15120766

**Published:** 2025-11-25

**Authors:** Ali H. Alghamdi, Mansuor A. Alanazi, Salwa Bukhari, Reham A. Alsumaira, Razan H. Alenzi, Abeer S. Aljuhani, Saud S. Alharbi, Mohammed A. Alsheikh

**Affiliations:** 1 Department of Radiological Sciences, Faculty of Applied Medical Sciences, University of Tabuk, Tabuk 71491, Saudi Arabia; 2 Department of Family and Community Medicine, Faculty of Medicine, University of Tabuk, Tabuk 71491, Saudi Arabia; menazi@ut.edu.sa (M.A.A.); salrehily@ut.edu.sa (S.S.A.); 3 Department of Radiology, Faculty of Medicine, University of Tabuk, Tabuk 71491, Saudi Arabia; s.bukhari@ut.edu.sa; 4 Department of Health Rehabilitation Sciences, Faculty of Applied Medical Sciences, University of Tabuk, Tabuk 71491, Saudi Arabia; 431000579@stu.ut.edu.sa (R.A.A.); 421002991@stu.ut.edu.sa (R.H.A.); 5 Diabetes Center, King Fahd Specialist Hospital, Tabuk 47717, Saudi Arabia; abeeraldebisi@hotmail.com; 6 Vascular and Interventional Radiology Department, King Fahad Specialist Hospital in Tabuk, Tabuk 47717, Saudi Arabia; moabalshaikh@moh.gov.sa

**Keywords:** Dual Energy X-ray Absorptiometry, type 2 diabetes, bone mineral density, body mass index

## Abstract

**Background/Objectives**: Type 2 diabetes mellitus (T2DM) is increasingly recognized as a contributor to skeletal fragility despite patients often having a normal or even elevated bone mineral density (BMD), a phenomenon described as the “T2DM bone paradox.” This study aimed to use DEXA screening to explore how metabolic and demographic factors, particularly body mass index (BMI), age, sex, and glycated hemoglobin (HbA1c), influence Bone Mineral Density (BMD) among Saudi adults, a population where diabetes and obesity are highly prevalent. **Methods**: A retrospective cross-sectional study was conducted among 89 adults (mean age 61.1 years; 82% female) who underwent dual-energy X-ray absorptiometry (DEXA) at King Fahad Specialist Hospital in Tabuk, Saudi Arabia. Bone mineral density was evaluated at the lumbar spine, femoral neck, and total hip. Correlation and multiple regression analyses were conducted to assess how age, sex, body mass index (BMI), and glycated hemoglobin (HbA1c) were related to BMD T-scores. **Results**: The prevalence of osteopenia and osteoporosis was 43.8% and 23.6%, respectively, with women and older adults showing the highest rates of low bone mass. Participants had a mean age of 61.1 ± 12.1 years, average BMI of 32 kg/m^2^, and mean HbA1c of 6.6 ± 1.8%. Females showed slightly lower T-scores at all skeletal sites compared with males (lumbar spine −1.81 vs. −1.55; femoral neck −1.15 vs. −0.76; total hip −0.62 vs. −0.12), indicating greater bone loss in women. BMI was consistently and positively associated with BMD across all skeletal sites (*p* < 0.05), whereas age and female sex were negative predictors at the femoral neck and hip. HbA1c showed a paradoxical positive relationship with BMD at weight-bearing sites, reflecting the complexity of metabolic effects on bone quality. The models explained up to 28% of the variance in BMD. **Conclusions**: Individuals with higher level BMI tended to have better bone mass, while older age and female sex were related to decreased BMD. The positive association between HbA1c and BMD supports the concept of the “diabetic bone paradox” and emphasizes the value of combining the evaluation of both metabolic and skeletal factors when assessing fracture risk in Middle Eastern populations.

## 1. Introduction

In recent decades, there has been a rising concern about type 2 diabetes mellitus (T2DM) among global health organizations, now considered one of the most common and challenging chronic diseases worldwide. Type 2 diabetes mellitus (T2DM) affects both physical and psychological health and imposes a substantial financial burden on healthcare systems [1,2,3]. According to the International Diabetes Federation [4], more than 537 million adults currently live with diabetes, and this number is expected to reach nearly 783 million by 2045. The concern is notably more pronounced in the Middle East and North Africa (MENA) region, where various socioeconomic and health-related trends may be contributing to its escalation, such as rapid urbanization, lifestyle changes, and increasing obesity rates, and thus intensifying the epidemic [5]. In Saudi Arabia, for instance, Recent evidence indicates that diabetes now affects more than 23% of the adult population, placing the nation among those with the highest prevalence rates worldwide [6].

Among the health complications associated with diabetes, osteoporosis and abnormalities in bone mineral density (BMD) are particularly concerning, yet they have not been thoroughly investigated. Bone disorders affecting skeletal strength are estimated to impact 30–50% of adults aged over 50 in Saudi Arabia [7]. There is a well-recognized intersection between metabolic and skeletal disorders. Exploring this link in greater depth could help develop better prevention and management strategies.

Dual-energy X-ray absorptiometry (DEXA) is generally regarded as the most reliable and established imaging technique for evaluating bone mineral density (BMD, g/cm^2^) at key skeletal sites such as the lumbar spine, femoral neck, and total hip [8,9,10,11]. DEXA outcomes are normally reported as T-scores, which makes comparisons of an individual’s bone density with the average of healthy young adults. According to WHO criteria (2023), T-scores between −1.0 and −2.5 indicate osteopenia, while values below −2.5 define osteoporosis, characterized by fragile bones and a higher fracture risk [12]. Understanding these outcomes can be particularly difficult in those affected by type 2 diabetes. This complexity likely stems from the combined effects of several metabolic and physiological processes. Despite normal or increased BMD, people with type 2 diabetes exhibit a greater tendency toward hip and spinal fractures [13,14].

This condition, described as the “diabetic bone paradox,” indicates that maintaining skeletal strength depends more on bone quality than on bone density alone. Chronic hyperglycemia contributes to this problem by promoting the formation of advanced glycation end-products (AGEs), which accumulate in bone collagen, weaken its structure, and increase fracture susceptibility [14,15]. At the same time, excess body weight places mechanical stress on the skeleton, stimulating osteoblast activity and increasing BMD [16,17]. Therefore, the combined effects of factors like BMI, age, sex, and glycemic control (HbA1c) make the link between diabetes and bone health highly complex and multifactorial [18,19].

Although DEXA is considered a reliable and widely available diagnostic tool, only a limited number of studies have used it to explored how bone mineral density in Saudi patients with type 2 diabetes relates to clinical variables such as BMI, HbA1c, age, and sex. In the absence of sufficient local evidence, clinicians tend to draw on international studies to explain this complexity, though such data may overlook regional variations. Without a contextual understanding, it is difficult to adequately monitor diabetic patients who are at higher risk of bone disorders and fractures. Timely identification of bone changes in diabetic patients supports better skeletal outcomes by reducing modifiable risks and allowing early therapeutic interventions.

The present study aims to fill this gap by examining bone health data obtained from DEXA scans of Saudi adults diagnosed with type 2 diabetes mellitus. It will also provide a report about the prevalence of osteopenia and osteoporosis; identify independent predictors of BMD at the lumbar spine, femoral neck, and total hip; and explore how sex, age, BMI, and HbA1c interact to influence bone outcomes. Interpreting the results through the framework of the diabetic bone paradox, the study provides locally relevant insights into the determinants of bone fragility in T2DM and supports the development of broader metabolic–skeletal screening strategies in Saudi Arabia.

## 2. Methods

### 2.1. Study Design and Population

In this retrospective cross-sectional study, we analyzed clinical records of DEXA measurements for patients who underwent dual-energy X-ray absorptiometry at King Fahad Specialist Hospital, Tabuk, Saudi Arabia, between January and December 2024. Patients were mainly referred to DEXA screening through the hospital’s endocrinology and metabolic bone units for bone health evaluation and fracture risk assessment. The study population consisted mainly of postmenopausal women and older men at risk for osteoporosis or fragility fractures.

Eligible participants were patients with complete DEXA scan results and corresponding anthropometric and laboratory data, including HbA1c values, available in the hospital’s electronic medical record. Exclusion criteria included secondary causes of osteoporosis (e.g., hyperparathyroidism, chronic kidney disease stage ≥ 3, malignancy), use of bone-modifying agents (bisphosphonates, denosumab, or glucocorticoids ≥ 5 mg/day prednisone equivalent within 12 months), or incomplete anthropometric or glycemic data.

A total of 89 patients (73 women and 16 men) met the inclusion criteria and were included in the final analysis. As a retrospective chart review, no interventions were performed.

Ethical approval was obtained from both the University of Tabuk Local Research Ethics Committee (Reference No. UT-689-313-2025) and the Institutional Review Board of King Fahad Specialist Hospital (National Committee of Bioethics Registration No. HAP-07-TU-001). A waiver of informed consent was granted, in accordance with national bioethics guidelines for minimal-risk retrospective studies.

DEXA was selected as the reference standard for non-invasive quantification of areal bone mineral density (aBMD) and diagnosis of osteopenia and osteoporosis. Because of its high sensitivity and reliability, it is considered the gold-standard method for evaluating bone health in both clinical practice and research [10].

All scans were performed and reviewed by board-certified radiologists specializing in musculoskeletal imaging, ensuring adherence to International Society for Clinical Densitometry (ISCD) technical standards for scan acquisition and analysis [20].

### 2.2. Clinical and Laboratory Assessments

Demographic and anthropometric data (age, sex, height, and weight) were extracted from clinical records at the time of DEXA scanning. The body mass index (BMI) for each patient was calculated using the usual equation: body weight in kilograms divided by height in meters squared (kg/m^2^). Type 2 diabetes mellitus (T2DM) was identified based on the American Diabetes Association (ADA) criteria, which defined as either a confirmed clinical diagnosis under active treatment or an HbA1c value of ≥6.5% obtained within three months of the DEXA scan [21,22]. HbA1c values were obtained from endocrine clinic and saved in patients’ records.

Because of the well-established link between adiposity and bone density, BMI was retained as a covariate in all regression analyses despite its limitations. DEXA-based body composition measures were not uniformly available; however, BMI served as a reliable surrogate of adiposity within this overweight cohort (mean BMI: 32.1 ± 6.7 kg/m^2^).

### 2.3. Bone Mineral Density Measurement Protocol

DEXA scans were performed using Hologic Discovery QDR series Dual Energy X-ray Absorptiometry (DEXA) Sanner. Scans were obtained and analyzed by certified technologists, then reviewed by radiologists to ensure the absence of artifacts, degenerative changes (e.g., aortic calcification, vertebral compression), or positioning errors that could spuriously elevate lumbar-spine readings. T-scores were calculated following ISCD recommendations—using the NHANES III reference database for femoral sites and the manufacturer’s young-adult reference for lumbar spine values [20].

Osteoporosis was defined according to World Health Organization (WHO) criteria as a T-score ≤ −2.5 at any of the three standard skeletal sites (lumbar spine L1–L4, femoral neck, or total hip) [23,24,25], given its biomechanical relevance and vulnerability to metabolic alterations.

### 2.4. Statistical Analysis

Data were analyzed using IBM SPSS Statistics version 25.0 (IBM Corp., Armonk, NY, USA). Continuous variables were summarized as means ± standard deviations (SD) or medians with interquartile ranges, depending on normality (assessed via Shapiro–Wilk tests and Q–Q plots). Between-group comparisons (e.g., male vs. female) employed independent *t*-tests for parametric data or Mann–Whitney U tests for non-parametric data. Categorical variables were compared using Chi-square or Fisher’s exact tests. In addition, we used Pearson or Spearman correlation coefficients to assess the associations between continuous predictors (age, BMI, HbA1c) and site-specific BMD T-scores. Correlation coefficients (r). In order to determine independent predictors of BMD, multiple linear regression models were developed for each site-specific T-score (lumbar spine, femoral neck, and total hip). Predictors included sex (female = 1), age, BMI, and HbA1c. Model diagnostics confirmed homoscedasticity, normality of residuals, and absence of multicollinearity (variance inflation factor < 2.5).

Because the sample size was relatively small (*n* = 89), the models were kept simple to avoid overfitting. Statistical significance was set at a two-tailed *p*-value of <0.05. In addition to *p*-values, β coefficients, 95% confidence intervals, and R^2^ values were reported to emphasize clinical relevance.

The methodological design—rooted in high-fidelity DEXA imaging, validated metabolic profiling, and radiologist-supervised quality assurance—was intended to clarify how age, sex, BMI, and glycemic control collectively shape skeletal integrity in T2DM.

This integrated dataset provided the foundation for the inferential analyses reported in the Results section, demonstrating the interplay between metabolic and mechanical determinants of bone health in a real-world clinical cohort.

## 3. Results

This study included 89 patients who had been referred from the endocrinology clinic for DEXA evaluation after being suspected or diagnosed with type 2 diabetes mellitus. The study cohort had an average age of 61.1 ± 12.1 years and consisted mainly of female participants (82%). The mean body mass index (BMI) was 32.1 ± 6.7 kg/m^2^, and the mean HbA1c was 6.6 ± 1.8%. Average bone mineral density (BMD) T-scores were −1.76 ± 1.51 for the lumbar spine, −1.08 ± 1.30 for the left femoral neck, and −0.53 ± 1.25 for the total left hip. Overall, these values indicate that most participants fell within the osteopenia range, particularly at the spine and femoral neck sites. Comparison of these values between males and females is also presented in Table 1.

### 3.1. Classification of Bone Status

In this study, 23.6% of patients (*n* = 21) were diagnosed with osteoporosis, 43.8% (*n* = 39) with osteopenia, while 32.6% (*n* = 29) had normal bone mineral density. The distribution of these categories by sex, age group, BMI, and glycemic control (HbA1c ≥ 6.5 %) is summarized in (Table 2).

**Figure 1:**
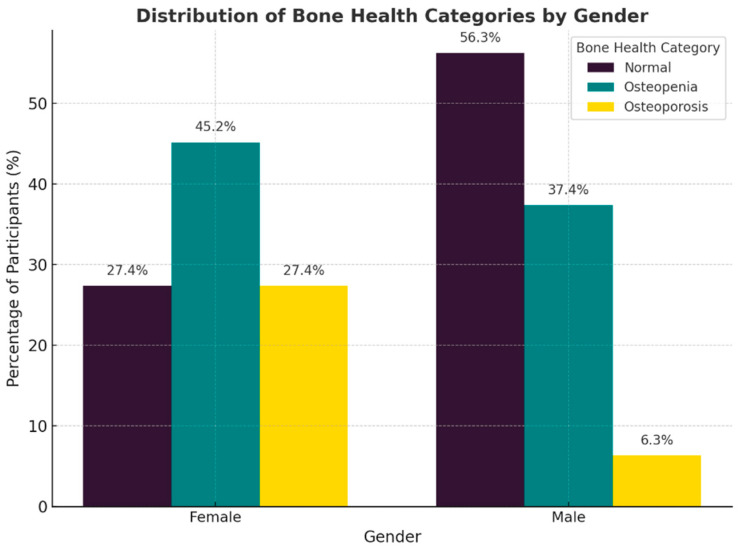
Distribution of Bone Health Categories by Gender. The graphic demonstrates the increased prevalence of low bone mass in women relative to males, highlighting distinct disparities in bone health profiles between the sexes.

**Figure 2:**
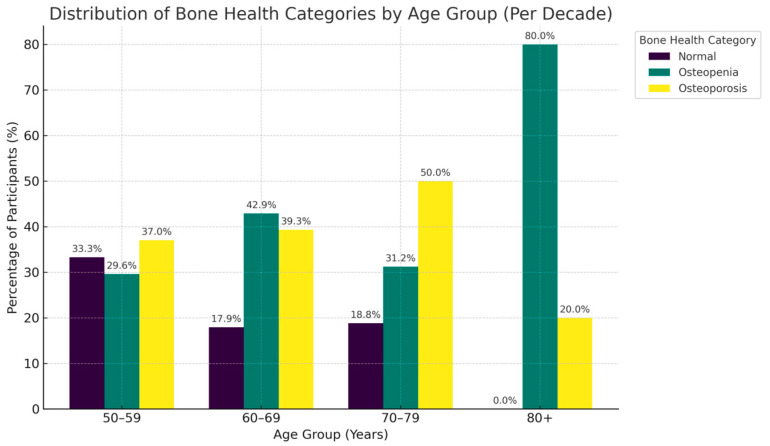
Distribution of Bone Health Categories by Age Group (Per Decade). This figure illustrates the age-related transition from normal bone mineral density in younger adults to higher prevalence rates of osteopenia and osteoporosis in older age groups.

**Figure 3:**
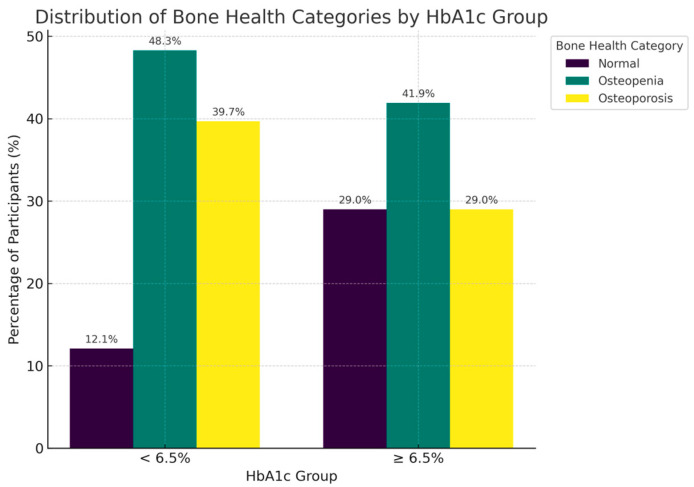
Distribution of Bone Health Categories by HbA1c. Distribution of bone health classifications based on HbA1c levels. The figure illustrates the relationship between glycemic control patterns and BMD classifications, demonstrating the metabolic intricacies of bone health in individuals with T2DM.

The distribution of bone health revealed marked differences across both gender (Figure 1),age groups (Figure 2) and glycemic control (Figure 3). Among women, osteopenia (45.2%) and osteoporosis (27.4%) were notably more common than in men. In men, the rates were 37.4% and 6.3%, respectively, with over 70% of women exhibiting low bone mass compared to less than half of men. Across age decades, bone deterioration followed a progressive trend, with osteopenia predominating in midlife (40–59 years) and osteoporosis increasing sharply beyond 60 years affecting half of individuals aged 70–79. Normal bone density was more frequent among younger patients and males, whereas osteoporosis was most common among older females, emphasizing how both age and sex contribute to bone weakness.

Moreover, osteoporosis was far less common in obese individuals (BMI ≥ 30 kg/m^2^; 18.4%) than in those with normal BMI (<25 kg/m^2^; 41.7%). Percentages derived from these smaller age groups should be interpreted with caution due to the limited number of participants in these categories. Interestingly, patients with poor glycemic control (HbA1c ≥ 6.5%) tended to have slightly higher BMD classifications, consistent with the positive correlations between HbA1c and T-scores reported later.

Classification based on the lowest T-score among the three skeletal sites.

### 3.2. Descriptive and Correlation Analyses

Female patients had slightly higher mean BMI (32.7 ± 6.9 kg/m^2^) than males (29.5 ± 5.2 kg/m^2^) but tended to exhibit lower T-scores across all skeletal sites.

Men showed higher mean femoral-neck (−0.76 ± 1.10 vs. −1.15 ± 1.34) and total-hip (−0.12 ± 0.86 vs. −0.62 ± 1.31) T-scores.

Correlation analyses demonstrated several significant relationships between demographic, metabolic, and densitometric variables (Table 3). BMI correlated positively with T-scores at the lumbar spine (r = 0.27, *p* = 0.01), femoral neck (r = 0.32, *p* = 0.003), and total hip (r = 0.26, *p* = 0.015). HbA1c also showed positive correlations with femoral-neck (r = 0.28, *p* = 0.008) and hip T-scores (r = 0.37, *p* < 0.001), indicating that higher HbA1c levels were paradoxically associated with higher BMD at weight-bearing sites.

Age correlated negatively with femoral-neck T-score (r = −0.21, *p* ≈ 0.05). Inter-site correlations among T-scores were strong (r = 0.65−0.77, *p* < 0.001).

### 3.3. Multivariable Regression Analyses

Three multiple linear regression models were constructed using T-scores at the lumbar spine, femoral neck, and total hip as dependent variables, with sex (female = 1), age, BMI, and HbA1c as predictors.

The findings are summarized in Table 4. In lumbar spine, only BMI remained a significant predictor (β = 0.061, *p* = 0.013), R^2^ = 0.10. And in femoral neck, female sex (β = −0.66, *p* = 0.045) and age (β = −0.030, *p* = 0.0048) were negative predictors, while BMI (β = 0.061, *p* = 0.0017) and HbA1c (β = 0.189, *p* = 0.0075) were positive; R^2^ = 0.26. In addition, in total hip, female sex (β = −0.71, *p* = 0.023) and age (β = −0.025, *p* = 0.010) were negative, while BMI (β = 0.045, *p* = 0.013) and HbA1c (β = 0.250, *p* < 0.001) were positive (R^2^ = 0.28).

### 3.4. Summary of Key Determinants

Across skeletal sites, our results show that BMI consistently demonstrated a protective association with BMD, remaining significant in all models. Age and female sex were significant negative predictors at appendicular sites (femoral neck and hip), while HbA1c showed a paradoxical positive association with BMD in these same regions. We also found that patients with higher HbA1c values tended to display fewer osteoporotic classifications (Table 2). Notably, the modest R^2^ values (0.10–0.28) suggest that other unmeasured factors may contribute to BMD variability in T2DM. Nevertheless, the present findings highlight the complex interplay between adiposity, glycemic status, and skeletal health in this population.

## 4. Discussion

In this cohort of 89 adults referred from a endocrine clinic undergoing dual-energy X-ray absorptiometry (DEXA) evaluation, body mass index (BMI) emerged as the most consistent independent determinant of bone mineral density (BMD) across all skeletal sites. Female sex and older age were associated with lower T-scores in weight-bearing regions, whereas glycated hemoglobin (HbA1c) demonstrated a paradoxical positive association with femoral-neck and total-hip BMD. Almost two-thirds of the patients were diagnosed with either osteopenia or osteoporosis, conditions that were more common among older and leaner women. These results emphasize the complex interaction between metabolic and demographic factors that shape bone health outcomes in individuals with type 2 diabetes mellitus.

### 4.1. BMI and Skeletal Protection in T2DM

The positive association between BMI and BMD observed in this study supports the mechanical loading hypothesis, where increased body weight places greater mechanical stress on the bones, thereby promoting bone formation [16,17]. In addition to mechanical effects, obesity might activate bone formation via endocrine mechanisms mediated by insulin, leptin, and estrogen [15]. Regionally, these findings align with Saudi and Gulf countries data. Alkhunizan et al. [26] and Aldukhayel [7] demonstrated higher BMD among overweight and obese Saudis compared with lean individuals. Several international studies have highlighted what is known as the “obesity–osteoporosis paradox.” They show that while individuals with obesity often have higher bone mineral density, this does not necessarily mean their bones are stronger or that their risk of fractures is lower [26,27,28]. These findings reinforce the consistency of BMI as a protective determinant of bone mass. This phenomenon can be explained by obesity-related metabolic changes, including chronic inflammation, insulin resistance, and fatty infiltration of bone tissue, which collectively impair bone microarchitecture. As a result, the skeleton may appear denser on imaging but is structurally compromised and more susceptible to fracture [29,30]. In agreement with global and local research, our results suggest that although a higher BMI is associated with increased BMD, it does not always translate into improved bone quality among individuals with diabetes.

### 4.2. Glycemic Control and the Diabetic Bone Paradox

An interesting finding of our study was the paradoxical positive correlation between HbA1c and BMD at the femoral neck and hip reflecting the “diabetic bone paradox,” where hyperglycemia coexists with higher BMD yet increased fracture risk [31,32]. Chronic hyperglycemia promotes the buildup of advanced glycation end-products (AGEs) in bone collagen, making the matrix stiffer but more brittle a paradox also observed among Middle Eastern populations [33,34,35]. According to Alshomar et al. [36], individuals with type 2 diabetes in Saudi Arabia exhibited increased lumbar spine BMD, even though fracture rates were notably high. Moreover, in Saudi Arabia, a hospital-based study reported higher lumbar BMD in T2DM versus non-diabetic women, with no consistent association between HbA1c and BMD, underscoring that hyperglycemia control does not straightforwardly map onto bone density [36]. Such agreement adds strength to the local evidence base. Collectively, these outcomes show that the paradox is consistently found among Saudi populations and is not only limited to Western-based research data. Such agreement supports its validity across different demographic settings. Mechanistically, hyperglycemia can disturb bone metabolism by reducing osteoblast formation and increasing oxidative stress, which together weaken normal bone remodeling [37,38]. Despite DEXA-detected higher density, these microarchitectural alterations may result in poorer bone quality and higher fragility fracture risk. In line with both global and regional studies, our findings confirm that patients with type 2 diabetes often present with normal or elevated bone mineral density, while their fracture risk may remain noticeably higher. This pattern has been reported in Asian [39], North American [40], and European [41] cohorts.

Although the Age and HbA1c interaction was examined, it was not statistically significant, indicating that the influence of glycemic control on age-related bone loss could not be confirmed in this sample. The prevalence of osteoporosis in our study (23.6%) aligns with national and regional estimates but is slightly higher than some previous Saudi findings. A recent national meta-analysis by the Saudi Osteoporosis Society (2023) reported an overall prevalence of 21.7% among older Saudi adults [42]. Differences in osteoporosis prevalence may be explained by variations in study populations, as our sample consisted mainly of older adults, women, and individuals with diabetes. It is also possible that other lifestyle factors such as diet, levels of sunlight exposure, and genetic background differ across Saudi regions and contribute to the variation seen in bone density [42,43,44]. The low R^2^ values indicate that age, sex, BMI, and HbA1c explain only a small part of the variation in BMD, suggesting that other unmeasured factors such as vitamin D status, calcium intake, physical activity, diabetes duration, and medication use likely account for some of the remaining variability, which is worth further investigation in future studies. Overall, our results emphasize the idea that DEXA interpretations should be considered within wider clinical and metabolic frameworks when assessing individuals with T2DM.

The interpretation of sex-related findings should be made with caution, as our sample was predominantly female. This imbalance limits the strength of male–female comparisons and may partly explain the apparent effect of female sex on BMD. With few male participants, the statistical power for sex-stratified analysis is reduced, making these results preliminary and less generalizable, particularly to male populations.

## 5. Limitations

The interpretation of our findings must be tempered by several limitations. The retrospective cross-sectional design prevents any causal inference and is vulnerable to unmeasured confounding variables. And the limited sample size, although sufficient for identifying significant effects, may have reduced our capacity to uncover more intricate associations, particularly in the logistic regression models for osteoporosis prediction. A further limitation is the lack of data on participants’ physical activity, an important determinant of bone metabolism and strength, which may have restricted comprehensive evaluation of behavioral influences on bone health. Additional key limitation of this study is the unequal sex distribution of the sample, with women representing the majority of the sample. This is due to the fact that these data were derived from existing medical records, which limited our ability to control the completeness and balance of the sample. This imbalance diminishes the statistical power for sex-stratified studies and constrains significant comparisons between men and women. As a result, confidence in sex-specific interpretations is reduced, and the generalizability of the findings—particularly for male populations—should be viewed with caution. Data on diabetes duration and medication use were not investigated, limiting evaluation of long-term and drug-related effects on bone health. Non-significant associations, particularly for HbA1c and the Age × HbA1c interaction, should be interpreted with caution, as the limited sample size may have reduced the power to detect subtle interaction effects. In addition, the reliance on areal BMD from DXA, while clinically standard, provides no direct insight into the microarchitectural deterioration such as compromised trabecular connectivity and cortical porosity that is believed to be central to diabetic bone disease. Furthermore, the absence of consistent DXA-derived body composition data hampered a more detailed examination of the unique effects of lean mass versus fat content on skeletal health.

## 6. Clinical Implications and Future Directions

Our research has significant implications for clinical practice in Saudi Arabia and other regions with a high prevalence of T2DM, despite these limitations. Firstly, it compellingly advocates for the incorporation of glycemic control metrics, primarily HbA1c, into the standard fracture risk evaluation of older persons, particularly postmenopausal women with Type 2 Diabetes Mellitus. A DXA scan analyzed without awareness of a patient’s diabetic condition or glucose history may yield an inadequate understanding of their skeletal susceptibility. Secondly, the femoral neck is a critical sentinel site that should be meticulously evaluated in diabetic patients, as our findings have demonstrated. Clinicians should be aware that a “normal” BMD at the lumbar spine may provide false reassurance if the femoral neck is compromised.

Future research must move beyond DXA. In order to identify the microarchitectural deficits that underlie the T2DM bone phenotype in this population, prospective, longitudinal studies that incorporate advanced imaging modalities such as trabecular bone score (TBS) and high-resolution peripheral quantitative computed tomography (HR-pQCT) are essential. Ultimately, there is a pressing need to develop and validate refined fracture risk algorithms—perhaps a FRAX-like tool augmented with diabetes-specific variables such as disease duration and HbA1c—to more accurately identify high-risk individuals for whom targeted preventive and therapeutic strategies can be deployed.

## 7. Conclusions

This study demonstrates that in Saudi adults with type 2 diabetes mellitus (T2DM), higher body mass index (BMI) is a consistent positive predictor of bone mineral density (BMD), while age and female sex are negative determinants. Interestingly, HbA1c was positively associated with BMD, illustrating the so-called “diabetic bone paradox,” in which greater bone density may not necessarily represent better bone health.

These results together highlight the importance of evaluating bone health in diabetic patients more broadly by combining metabolic indicators with DXA findings and also considering other lifestyle factors. Early screening, especially for older adults and women, may help reduce the risk of fragility fractures in this population. Further, multicenter studies are needed to examine bone quality and microstructural changes, which may help explain the mechanisms responsible for this paradox.

## Figures and Tables

**Table 1:** Descriptive characteristics of the study population. This is a summary of the study cohort’s densitometric, metabolic, and demographic traits. Age, BMI, HbA1c, and average T-scores for each skeletal site for the entire sample are displayed in the table, along with comparisons between males and females.

Variable	Total (*n* = 89)	Females (*n* = 73)	Males (*n* = 16)
Age (years)	61.1 ± 12.1	60.6 ± 11.5	63.3 ± 15.1
BMI (kg/m^2^)	32.1 ± 6.7	32.7 ± 6.9	29.5 ± 5.2
HbA1c (%)	6.6 ± 1.8	6.6 ± 1.9	6.6 ± 1.4
T-score lumbar spine	−1.76 ± 1.51	−1.81 ± 1.51	−1.55 ± 1.51
T-score femoral neck	−1.08 ± 1.30	−1.15 ± 1.34	−0.76 ± 1.10
T-score total hip	−0.53 ± 1.25	−0.62 ± 1.31	−0.12 ± 0.86

**Table 2:** Classification of bone status (normal, osteopenia, osteoporosis) by demographic and metabolic variables. This is a classification of participants into normal BMD, osteopenia, and osteoporosis groups, categorized by sex, age groups, BMI categories, and HbA1c levels. The table emphasizes differences in bone health trends among various demographic and metabolic subgroups.

Variable	*n*	Normal BMD *n* (%)	Osteopenia *n* (%)	Osteoporosis *n* (%)
Total	89	29 (32.6%)	39 (43.8%)	21 (23.6%)
Sex				
Female	73	20 (27.4%)	33 (45.2%)	20 (27.4%)
Male	16	9 (56.3%)	6 (37.4%)	1 (6.3%)
Age group				
40–49 years	10	1 (10.0%)	8 (80.0%)	1 (10.0%)
50–59 years	27	9 (33.3%)	8 (29.6%)	10 (37.0%)
60–69 years	28	5 (17.9%)	12 (42.9%)	11 (39.3%)
70–79 years	16	3 (18.8%)	5 (31.2%)	8 (50.0%)
80+	5	0 (0.0%)	4 (80.0%)	1 (20.0%)
BMI category				
<25 kg/m^2^	12	2 (16.7%)	5 (41.7%)	5 (41.7%)
25–29.9 kg/m^2^	28	6 (21.4%)	15 (53.6%)	7 (25.0%)
≥30 kg/m^2^	49	21 (42.9%)	19 (38.8%)	9 (18.4%)
HbA1c category				
<6.5%	58	7 (12.1%)	28 (48.3%)	23 (39.7%)
≥6.5%	31	9 (29.0%)	13 (41.9%)	9 (29.0%)

**Table 3:** Pearson correlation coefficients among continuous variables. This table shows the associations among age, BMI, HbA1c levels, and site-specific T-scores. The table offers a comprehensive overview of the relationships between metabolic and demographic variables and bone mineral density at the lumbar spine, femoral neck, and total hip.

Variable	Age	BMI	HbA1c	T-Score (L-Spine)	T-Score (Femoral Neck)	T-Score (Total Hip)
Age	1	0.04	0.15	0.01	−0.21	−0.16
BMI		1	0.18	0.27 *	0.32 **	0.26 *
HbA1c			1	0.16	0.28 **	0.37 ***
T-score (Spine)				1	0.65 ***	0.53 ***
T-score (Neck)					1	0.77 ***
T-score (Hip)						1

* *p* < 0.05; ** *p* < 0.01; *** *p* < 0.001.

**Table 4:** Multivariable linear regression models predicting BMD T-scores. Multivariable regression models identifying independent predictors of T-scores at the lumbar spine, femoral neck, and total hip. The table delineates the influence of sex, age, BMI, and HbA1c on BMD, encompassing regression coefficients and the performance metrics of the model.

Outcome	Predictor	β (95% CI)	*p*-Value	R^2^
Lumbar spine	Female (1)	−0.46 (−1.28, 0.36)	0.27	0.10 (0.06)
	Age (per year)	−0.004 (−0.03, 0.02)	0.76	
	BMI (per kg/m^2^)	0.061 (0.013, 0.109)	0.013	
	HbA1c (per %)	0.096 (−0.078, 0.270)	0.28	
Femoral neck	Female (1)	−0.66 (−1.31, −0.01)	0.045	0.26 (0.22)
	Age	−0.030 (−0.050, −0.009)	0.0048	
	BMI	0.061 (0.024, 0.099)	0.0017	
	HbA1c	0.189 (0.052, 0.326)	0.0075	
Total hip	Female (1)	−0.71 (−1.32, −0.10)	0.023	0.28 (0.24)
	Age	−0.025 (−0.045, −0.006)	0.010	
	BMI	0.045 (0.010, 0.081)	0.013	
	HbA1c	0.250 (0.120, 0.380)	<0.001	

## Data Availability

The data supporting this study’s findings are available from the corresponding author upon reasonable request. Data are not publicly available due to privacy and ethical restrictions.
